# Shaping the import system of mitochondria

**DOI:** 10.7554/eLife.38209

**Published:** 2018-06-20

**Authors:** Kostas Tokatlidis

**Affiliations:** Institute of Molecular Cell and Systems BiologyUniversity of GlasgowGlasgowUnited Kingdom

**Keywords:** mitochondria, trypanosome, outer membrane, MIM complex, convergent evolution, TOM complex, *S. cerevisiae*

## Abstract

Evidence is accumulating that unrelated species have independently evolved the same way of importing proteins in their mitochondria.

**Related research article** Vitali DG, Käser S, Kolb A, Dimmer KS, Schneider A, Rapaport D. 2018. Independent evolution of functionally exchangeable mitochondrial outer membrane import complexes. *eLife* **7**:e34488. doi: 10.7554/eLife.34488

Mitochondria are organelles that fulfil a variety of critical functions in eukaryotic cells, and the event that resulted in their creation two billion years ago – when a bacterium fused with an ancient cell – was a defining moment in the evolution of life (Gray et al., 1999). However, the mitochondrial genome encodes a mere 13 different polypeptides, so the vast majority of the roughly 1500 mitochondrial proteins are made in the cytosol, and then imported into the organelle. These proteins are recognized and processed by various complexes which are embedded in the two membranes (the inner and outer mitochondrial membrane) that enclose a mitochondrion (Schmidt et al., 2010; Dolezal et al., 2006).

The import proteins present in the mitochondrial membranes can fold to form one of two structures: an α-helix or a β-barrel. How β-barrel proteins are taken into the mitochondrial outer membrane in the first place has been studied in much detail, and this process requires the translocase complex of the outer membrane, or TOM, to work with a structure called SAM (sorting and assembly machinery). The TOM complex is thought to be the main entrance for all mitochondrial proteins, irrespective of their final location within the organelle.

By contrast, it is less clear how α-helix proteins are brought to the outer membrane of mitochondria. However, several studies have suggested that, in fungi, a third ‘MIM’ (for mitochondrial import machinery) complex is involved (Figure 1; Becker et al., 2008). So far, it is known that Mim1 and Mim2 – the two proteins that form the MIM complex – are present in fungi but not in other eukaryotes.

**Figure 1.**
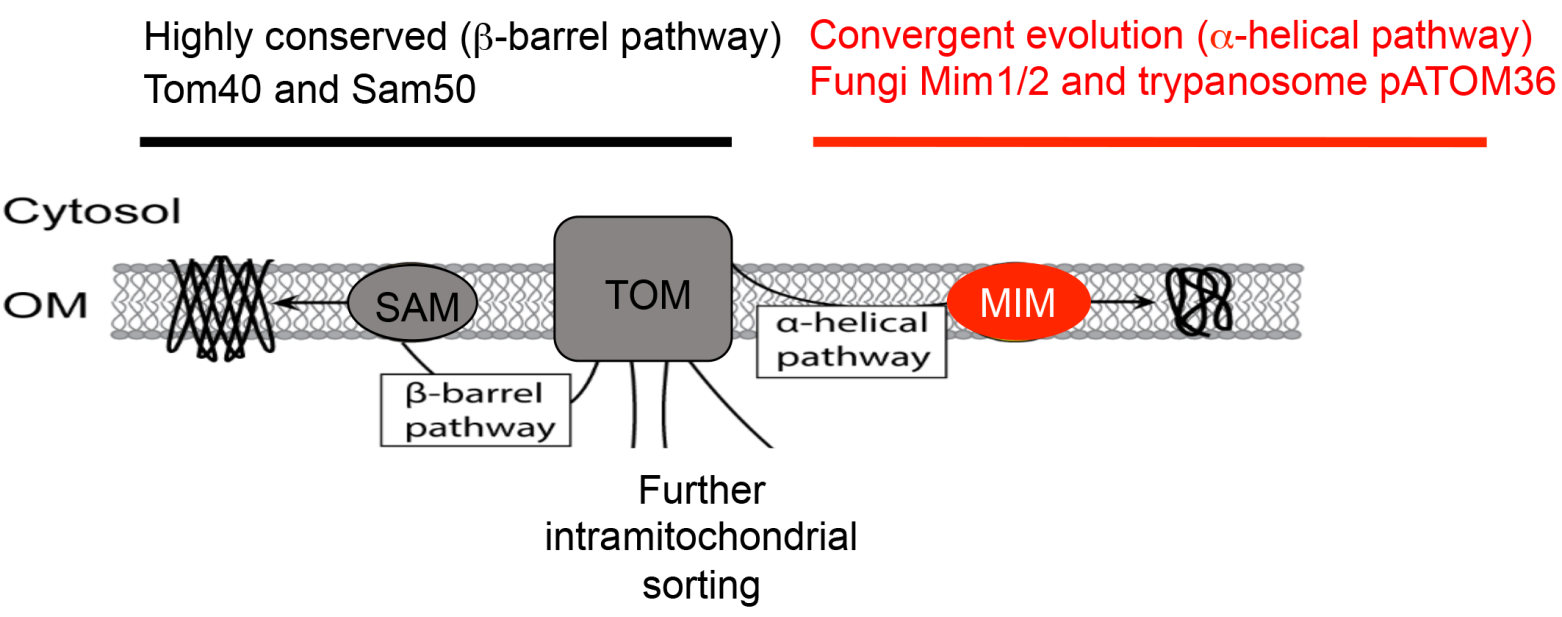
Protein import complexes in mitochondria. The outer mitochondrial membrane (OM) contains embedded protein complexes – such as the SAM, TOM and MIM complexes – that import proteins from the cytosol into the mitochondria. The SAM and TOM complexes interact to import β-barrel proteins (left). Certain subunits in the complexes (Tom22, Tom40 and Sam50) are highly conserved in all eukaryotes. However, the MIM complex, which imports α-helix proteins (right), is only present in fungi. Vitali et al. now show that pATOM36, an import protein found in the trypanosome *T. brucei*, and the MIM complex are functionally equivalent, despite their sequences being very different. This presents an exciting case of convergent evolution in a core protein import machinery of mitochondria. TOM: translocase complex of the outer membrane; SAM: sorting and assembly machinery; MIM: mitochondrial import machinery.

IMAGE CREDIT: Dr Afroditi Chatzi.

Biochemical and genome analyses of the TOM and SAM complexes across different organisms show that only a few subunits (Tom22, Tom40 and Sam50) are conserved in all eukaryotes. It is likely that the protein import system in the bacteria that became the modern mitochondria was made from these subunits. Other subunits are not conserved: for example, sequence analyses of two subunits of the TOM complex, Tom20 and Tom70, indicated that they evolved separately in fungi and plants. However, structural experiments showed that these subunits have adopted common structures that allow them to recognize and import mitochondrial proteins (Perry et al., 2006).

This was the first time a process known as convergent evolution – when species that are not related independently evolve similar structures to perform identical roles – had been observed in the mitochondrial import system. Further studies revealed that the trypanosome *T. brucei* also has receptors that have evolved separately from those in fungi and animals, but then converged to perform the same role (Mani et al., 2015). Now, in eLife, Doron Rapaport of the University of Tübingen, André Schneider of the University of Bern, and colleagues – Daniela Vitali, Sandro Käser and Antonia Kolb (as joint first authors), and Kai Dimmer – report another exciting example of convergent evolution, this time not for accessory receptor subunits but for a core import complex (Vitali et al., 2018).

In *T. brucei*, a protein called pATOM36 is found in the outer membrane of the mitochondria, where it helps to import other proteins. It is not related to the Mim1 receptor found in fungi, and their sequences are very dissimilar, but Vitali et al. have found that fungi in which the MIM complex has been replaced with pATOM36 can still import proteins. However, pATOM36 is not as effective as Mim1, possibly because it has evolved to prefer substrates that are only found in trypanosomes.

Likewise, Vitali et al. show that the MIM complex can take the place of pATOM36 in trypanosomes, providing that Mim1 and Mim2 are expressed at approximately the same levels. These largely unexpected results suggest that the MIM complex and pATOM36 perform their roles alone; indeed, it is unlikely that they could have found molecular partners to work with when placed in an unfamiliar environment.

How can MIM and pATOM36 replace each other when they are so distantly related? Both are embedded in the outer mitochondrial membrane, and are formed of several subunits, but the exact topology of pATOM36 is unknown. Structural analyses may provide important clues because a similarity in their structure could explain the overlap in their function. This would not be unprecedented; for example, proteins found in yeast mitochondria and bacteria fold into similar structures that allow them to bind to the same types of molecules (Alcock et al., 2008).

Another possibility is that MIM and pATOM36 have distinct structures that work in different ways but reach the same outcome – and attach to the same proteins. Again this would not be unprecedented; enzymes present in yeast and bacteria can use distinct mechanisms to create identical chemical links known as disulfide bonds in proteins (Riemer et al., 2009).

The work of Vitali et al. provides an intriguing hint that convergent evolution may have added components to the ancestral core import machinery in a modular way. In the future, biochemical, structural and genomics analyses of distant species could be combined to provide interesting clues, and maybe some surprises, about the evolution of the protein import systems of mitochondria. These answers may help us understand how an ancestral bacterium morphed into the organelle that powers most eukaryotic species today.
